# Daily Evaluation of COVID-19 Patients Primarily Based on Lung Ultrasound: In Times of Emergency, It's Time to Change Some Paradigms

**DOI:** 10.4269/ajtmh.20-0596

**Published:** 2020-06-12

**Authors:** Francesco Palmese, Barbara Caroli, Alessandro Graziani, Grazia Zanframundo, Rossella Del Toro, Elisabetta Sagrini, Pierluigi Cataleta, Marco Domenicali

**Affiliations:** AUSL della Romagna; Department of Internal Medicine; S. Maria delle Croci Hospital; Ravenna, Italy; E-mails: francesco.palmese@auslromagna.it, barbara.caroli2@auslromagna.it, alessandro.graziani@auslromagna.it, grazia.zanframundo@auslromagna.it, rossella.deltoro@auslromagna.it, elisabetta.sagrini@auslromagna.it, pierluigi.cataleta@auslromagna.it; AUSL della Romagna; Department of Internal Medicine; S. Maria delle Croci Hospital; Ravenna, Italy; Department of Medical and Surgical Sciences; University of Bologna; Bologna, Italy; E-mail: m.domenicali@unibo.it

Dear Sir,

As physicians accustomed to daily evaluation of patients with pneumonia with lung ultrasound (LUS) and working in an area (northern Italy) with a high incidence of COVID-19, we read with great interest the retrospective observational study in the *American Journal of Tropical Medicine and Hygiene* by Yasukawa and Minami^1^ about the potential use of LUS to evaluate SARS-CoV-2 pneumonia.

In fact, from the beginning of the SARS-CoV-2 pandemic, we realized that, to deal with this epochal challenge, we would need to rethink some cornerstones of our daily clinical practice, particularly in regard to the daily evaluation of these patients.

As is well known, the diagnostic path to assess patients requires comprehensive consideration of exposure history, clinical manifestations, laboratory tests, and imaging examinations.^2^

High-resolution computed tomography of the chest represents the gold standard to diagnose SARS-CoV-2–related pneumonia.^2^ However, the use of chest computed tomography (CT) has several limitations: it is expensive, impractical for high numbers of patients, and entails radiation exposure. Thus, despite its key role as a diagnostic tool, chest CT is not feasible for frequent monitoring of patients during hospitalization.

In our daily clinical practice, we have been accustomed to use stethoscope auscultation as the main tool to evaluate patients with lung disease, to monitor the clinical evolution of these patients, and to exclude complications such as bacterial pneumonia and acute heart failure. However, in the management of patients with COVID-19, auscultation is limited by extensive personal protective equipment and requires close contact with a potentially infectious patient. We have to reach the right balance between ensuring an adequate level of patient monitoring and reducing exposure of clinicians, to limit the spread of the epidemic and to not undermine the healthcare system.

Several studies have demonstrated that LUS has comparable or superior accuracy compared with chest radiography for many of the most common causes of dyspnea.^3^ However, few studies have compared LUS with pulmonary auscultation^4^ in the follow-up of patients.

In light of the aforementioned, we have decided to use LUS as the main tool to daily evaluate patients with SARS-CoV-2–related pneumonia. Lung ultrasound benefits from its good diagnostic accuracy, short execution time, and limited necessary contact with patients.

During the first week of the epidemic at our hospital, we performed both detailed stethoscope auscultation and LUS in all patients who had an interstitial pneumonia diagnosed by chest CT on admission. After this first week, we decided to monitor our patients only with LUS, with examinations every other day using a systematic approach tailored to specific patients, and focusing on the posterior and lateral regions, where pathological findings were mainly located by chest CT. We performed a retrospective evaluation of 66 patients admitted for SARS-CoV-2–related pneumonia at the beginning of the epidemic at our middle-intensity ward (“S. Maria delle Croci” Hospital, Ravenna, Italy). Demographic and clinical features of patients are summarized in Table 1. During the first week, auscultation identified the presence of lung sounds such as crackles only in a small number of patients (18/66, 27%), but, with LUS, we found reverberation artifacts (B-lines) in almost all patients (63/66, 95%), with focal, multifocal, and diffuse patterns. In some patients, an irregular pleural line with small subpleural confluent consolidations was described; in almost all patients, some spared areas, mixed with pathological areas, were present bilaterally.

**Table 1:** Demographic and clinical features of patients (*n* = 66)

Male gender, *n* (%)	36 (55)
Age (years), mean (SD)	58 (12)
Symptoms at admission, *n* (%)	
Fever	62 (94)
Cough	55 (83)
Dyspnea	22 (33)
Asthenia	25 (38)
Arterial blood gas analysis at admission	
PaO_2_ (mmHg), mean (SD)	77 (13)
SaO2 (%), mean (SD),	95^2^
PaO_2_/FiO_2_ (P/F) ratio (mmHg), mean (SD)	352 (12)
High-resolution computed tomography at admission, *n* (%)	
Ground-glass opacity pattern	63 (95)
Consolidation pattern	24 (36)
Ultrasound findings, *n* (%)	
B-lines	45 (68)
Subpleural confluent consolidations	7 (11)
Stethoscope auscultation findings, *n* (%)	
Crackles	18 (27)
Non-pathological findings	48 (73)

PaO_2_ = partial pressure of arterial oxygen; FiO_2_ = fraction of inspired oxygen; SaO_2_ = arterial oxygen saturation.

**Figure 1.**
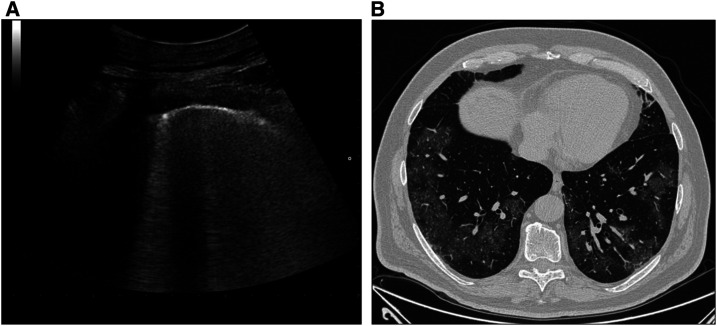
(**A**) Ultrasound scan of the right lung parenchyma using the convex transducer that shows B-lines. (**B**) Axial high-resolution computed tomography image of the same region showing bilateral and diffuse ground-glass opacities.

Lung ultrasound findings showed strong correlation with CT findings in terms of localization and degree of lung involvement (Figure 1). Furthermore, when chest CT was repeated to check the evolution of findings in a subgroup of patients, it confirmed improvement that had been documented by LUS. Finally, after we discontinued the use of stethoscope auscultation, when an improvement was documented with LUS, it always corresponded with clinical improvement.

We are experiencing an increasing interest in LUS in patients with COVID-19 pneumonia, as demonstrated by the growing number of studies on this specific topic.^5,6^ However, these studies mainly focus on the potential role of LUS as a diagnostic tool for initial assessment or as a monitoring tool in high-intensity settings.^7^

Our experience suggests that an ultrasound-driven approach in a middle-intensity setting may be appropriate for the daily management of patients affected by SARS-CoV-2 pneumonia. Stethoscope auscultation appears to be not as informative as LUS in this specific setting. Moreover, LUS offers a relatively safe diagnostic bed-side test, minimizing the risk of infection of caregivers. As evidence for this, none of the physicians on our team has developed a SARS-CoV-2 infection more than 3 months after the onset of the epidemic. From a clinical perspective, we are strongly satisfied with the ability of LUS to provide signs of worsening lung conditions and to help us identify patients at major risk of clinical deterioration and, thus, in need of prompt transfer to an intensive care unit.

In conclusion, we strongly recommend the use of LUS in the daily evaluation of COVID-19 patients because, based on our experience, it is inexpensive, quick to perform, more sensitive than auscultation for identification of worsening lung disease, and safer for caregivers.
